# Case report: A challenging case of stage IVB mixed neuroendocrine-non-neuroendocrine neoplasm of the gallbladder treated with extended radical resection including portal vein reconstruction

**DOI:** 10.3389/fonc.2026.1834076

**Published:** 2026-06-08

**Authors:** Quanlei Wang, Shun Ruan, Xiyin Ye, Haifeng Wang, Quli Zeng, Xiao Luo, Kai Guo, Ailing Zhang, Huixiang Huang, Gang Wu, Haibo Chen, Suhui Zeng, Tiansheng Xie, Wanhong Liang, Weihong Duan, Sangui Wang

**Affiliations:** 1 Department of Surgery, Dongguan Nancheng Hospital, Dongguan, China; 2 Dongguan Institute of Gallbladder Disease Research, Dongguan Nancheng Hospital, Dongguan, China; 3 Department of Critical Care Medicine, Dongguan Nancheng Hospital, Dongguan, China; 4 Department of Pathology, Dongguan Nancheng Hospital, Dongguan, China; 5 Department of Rehabilitation Medicine, Dongguan Nancheng Hospital, Dongguan, China; 6 Department of Medical Administration, Dongguan Nancheng Hospital, Dongguan, China; 7 Department of Laboratory, Dongguan Nancheng Hospital, Dongguan, China; 8 Department of Radiology, Dongguan Nancheng Hospital, Dongguan, China; 9 Department of Hepato-Biliary-Pancreatic Surgery, People's Liberation Army (PLA) Rocket Force Characteristic Medical Center, Beijing, China

**Keywords:** gallbladder carcinoma, large cell neuroendocrine carcinoma, mixed neuroendocrine-non-neuroendocrine neoplasms, multidisciplinary management, pancreaticoduodenectomy

## Abstract

**Background:**

Gallbladder cancer is a highly lethal biliary tract malignancy. Among them, mixed neuroendocrine-non-neuroendocrine neoplasms (MiNEN), particularly those containing large cell neuroendocrine carcinoma (LCNEC) components, are extremely rare and highly aggressive. Due to its low incidence and insidious symptoms, reports on the clinicopathological characteristics and optimal treatment strategies for such tumors are very limited, with diagnosis primarily relying on pathology and immunohistochemistry.

**Case presentation:**

A 78-year-old female was incidentally found to have a gallbladder mass during a routine physical examination, accompanied by a significantly elevated carcinoembryonic antigen (CEA) level (84.09 ng/ml). MRI imaging indicated gallbladder malignancy with multiple liver and hilar lymph node metastases. The patient underwent extended radical surgery, including partial hepatectomy, pancreaticoduodenectomy, and partial portal vein resection with anastomosis. Postoperative pathology and immunohistochemistry confirmed a mixed adenoma-large cell neuroendocrine carcinoma of the gallbladder with multiple metastases. Immunohistochemistry showed Syn (+), CK19 (+), Ki-67 (+) approximately 80%, INSM1 (+), and CgA (+) approximately 10%. All surgical margins were negative. The patient recovered well postoperatively.

**Conclusion:**

This case report describes a rare case of locally advanced mixed adenoma-large cell neuroendocrine carcinoma of the gallbladder in primary hospital. Achieving negative margins through aggressive extended radical surgery may be a feasible treatment option in carefully selected patients; however, the oncological benefit remains to be validated. It emphasizes the critical role of multidisciplinary collaboration in diagnosis and treatment and adds new data to the limited literature on this rare disease.

## Introduction

Gallbladder cancer is the most common biliary tract malignancy, yet it remains a relatively rare and highly lethal cancer, often diagnosed at an advanced stage due to its insidious onset and non-specific symptoms (1). The vast majority of gallbladder cancers are adenocarcinomas (2). Neuroendocrine neoplasms originating in the gallbladder are extremely rare, accounting for less than 0.5% of all gallbladder tumors and representing only a small fraction of all gastrointestinal neuroendocrine neoplasms (3). Among these, large cell neuroendocrine carcinoma (LCNEC) is an exceedingly rare and aggressive subtype, characterized by high-grade malignant potential and poor prognosis (3). Mixed tumors containing both adenocarcinoma and large cell neuroendocrine carcinoma components are even rarer; according to the 2022 WHO Classification of Digestive System Tumours, such tumors are classified as mixed neuroendocrine-non-neuroendocrine neoplasms (MiNEN) (4). The diagnosis of these tumors relies heavily on histopathological examination and immunohistochemical staining, where markers such as synaptophysin (Syn), INSM1, and a high Ki-67 proliferation index are key to confirming neuroendocrine differentiation and grade (3). Due to their rarity and aggressiveness, there is currently no standardized treatment protocol, although radical surgical resection with negative margins remains the only potential pathway to long-term survival (2–4). This case report provides new insights into the clinical presentation, pathological features, and surgical management of a locally advanced gallbladder mixed adenoma-large cell neuroendocrine carcinoma, contributing to the limited literature in this field.

## Case report

### Preoperative comprehensive examination

**Figure 1:**
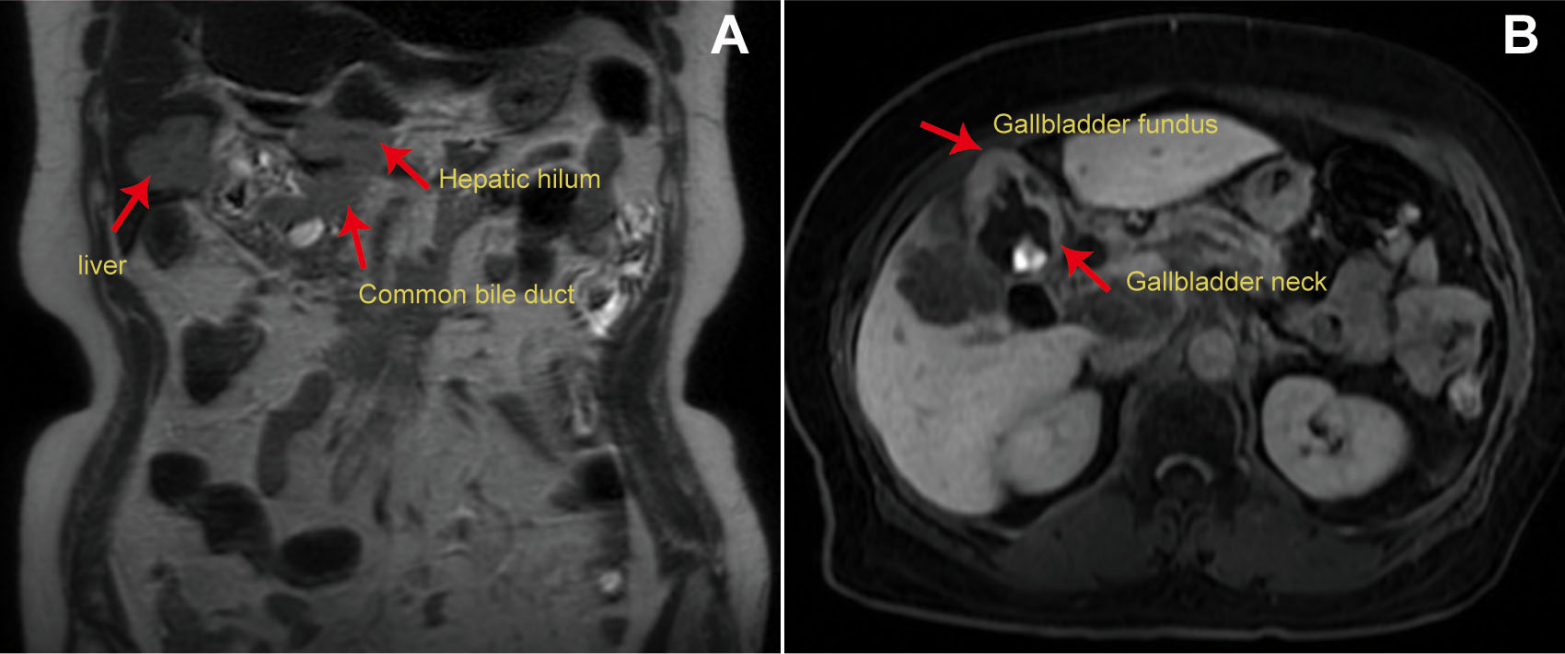
Upper abdominal MRI showing gallbladder carcinoma with metastases to the liver, hepatic hilum, and multiple other sites; **(A)** Coronal view, **(B)** Axial view.

A 78-year-old female presented to our outpatient clinic due to the incidental finding of gallbladder and liver space-occupying lesions on ultrasound during a routine physical examination. Physical examination revealed no abnormalities. Laboratory blood tests showed a carcinoembryonic antigen (CEA) level of 84.09 ng/ml, elevated total cholesterol (CHO) at 5.37 mmol/L and low-density lipoprotein cholesterol (LDL-C) at 3.87 mmol/L, and decreased high-density lipoprotein cholesterol (HDL-C) at 1.25 mmol/L. Other indicators showed no significant abnormalities. Further MRI examination revealed an enlarged gallbladder with multiple nodular signal intensities within the lumen, irregular thickening of the wall in the body and fundus of the gallbladder, and irregular large patchy abnormal signals in the adjacent liver parenchyma. Multiple enlarged lymph nodes were observed in the porta hepatis, which merged into massive nodules and showed ring enhancement on contrast-enhanced scans. These findings were suggestive of gallbladder malignancy with multiple metastases to the liver and porta hepatis (**Figure 1**).

### Complex multivisceral resection and multidisciplinary management

The patient was extremely reluctant to undergo standard preoperative chemotherapy due to concerns about delays and side effects, and strongly desired immediate surgery. After multidisciplinary team (MDT) discussion, it was decided to administer three days of fluorouracil (0.5 g qd) preoperatively, followed directly by radical resection including partial hepatectomy (segments S4b/S5), pancreaticoduodenectomy, partial portal vein resection with anastomosis, and cholecystectomy.

The surgical procedure was performed under general anesthesia. An upper abdominal reverse “L”-shaped incision was made. Intraoperative findings confirmed an enlarged gallbladder with irregularly thickened walls densely adhered to surrounding tissues, hard masses in liver segments S4b/S5, and enlarged fused hilar lymph nodes encasing the portal vein over approximately 2.5 cm. The distal stomach (~25%) was transected using a linear stapler. The pancreas was transected with a Harmonic scalpel, and an F8 stent tube was placed in the main pancreatic duct. The duodenum and pancreatic head were mobilized medially, exposing the inferior vena cava and aorta. The proper hepatic artery, common bile duct, and portal vein were skeletonized. The jejunum was divided 10 cm distal to the Treitz ligament.

Liver parenchymal transection of segments S4b/S5 was performed under temporary portal vein occlusion using a Harmonic scalpel, with careful preservation of the middle hepatic vein. The specimen (including the gallbladder and resected liver segments) was removed. The involved portal vein segment was resected after systemic heparinization and vascular clamping; an end-to-end anastomosis was performed using 5-0 Prolene sutures (anastomosis time 12 minutes). No significant liver congestion was observed. The procedure was completed with pancreaticojejunostomy, choledochojejunostomy, and gastrojejunostomy. Intraoperative blood loss was approximately 400 mL, total surgical time was approximately 3 hours.

**Figure 2:**
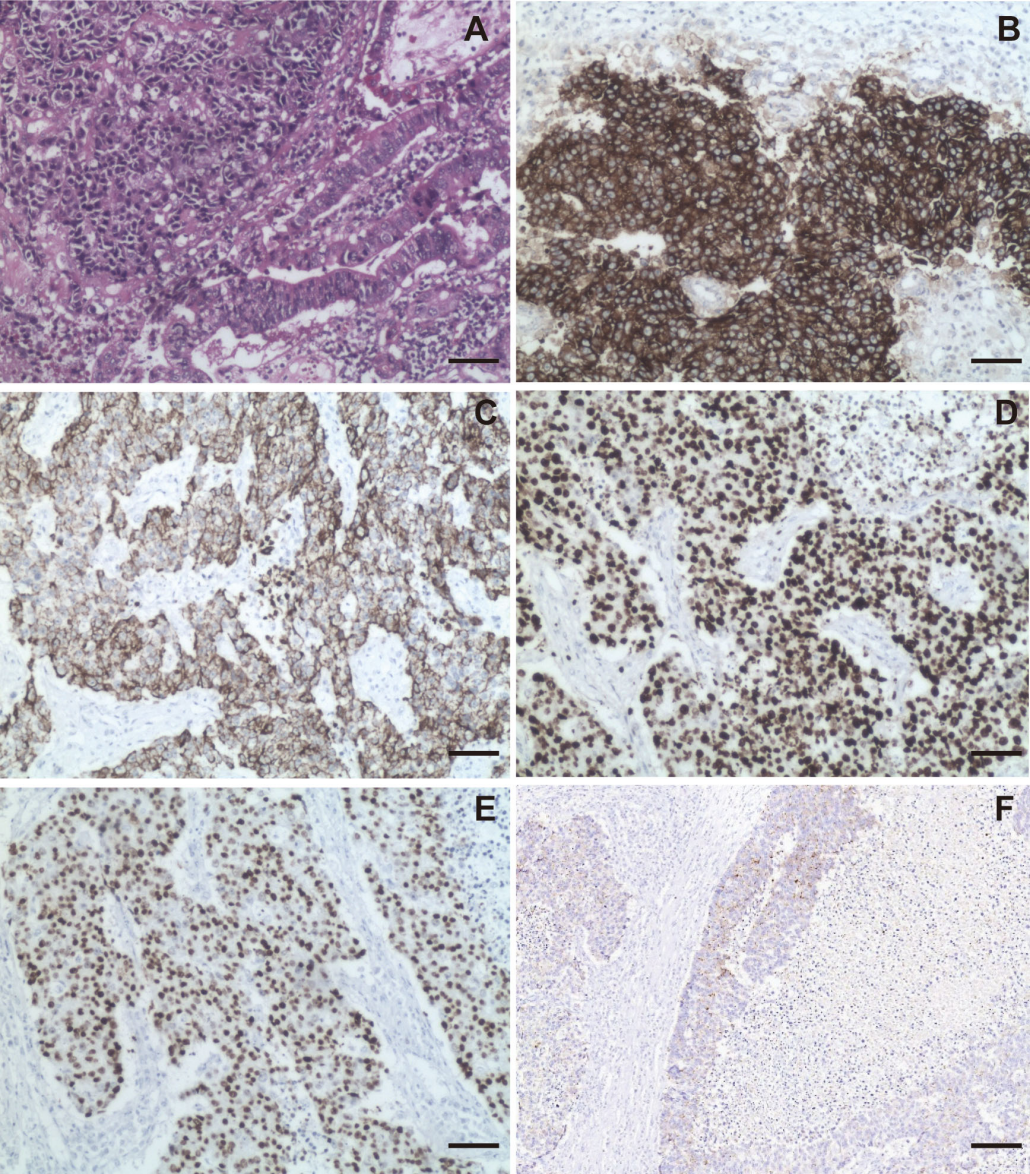
Histopathological and immunohistochemical characteristics of the tumor. **(A)** Hematoxylin and eosin (HE) staining reveals the coexistence of approximately 60% adenocarcinoma and 40% large cell neuroendocrine carcinoma components (×100). Tumor cells demonstrate strong immunoreactivity for **(B)** synaptophysin (Syn, cytoplasmic, ×100), **(C)** cytokeratin 19 (CK19, membranous, ×100), and **(D)** Ki-67 (nuclear, approximately 80%, ×100), **(E)** INSM1(nuclear, ×100), and **(F)** chromogranin A (CgA, cytoplasmic, approximately 10%, ×100).

Postoperative routine histopathological examination and immunohistochemistry results showed: tumor cells were positive for Syn (+), CK19 (+), Ki-67 (+, approximately 80%, INSM1(+), and CgA (+, approximately 10%) (**Figure 2**). The morphological features of tumor cells in the gallbladder tumor, liver tumor, hilar tumor, and common bile duct tumor were essentially identical. The tumor at the gallbladder fundus was a moderately-to-poorly differentiated carcinoma, consistent with mixed adenocarcinoma-large cell neuroendocrine carcinoma, invading the serosal layer of the gallbladder. Perineural invasion was observed, and no intravascular tumor emboli were seen. The maximum tumor diameter was 8 cm. The tumor at the gallbladder neck was a poorly differentiated carcinoma with a maximum diameter of 4 cm, surrounded by lymphoid tissue, consistent with metastatic large cell neuroendocrine carcinoma. The liver tumor and hilar tumor were poorly differentiated carcinomas, with maximum diameters of 10 cm and 8 cm, respectively. They appeared as multiple nodules with focal patchy necrosis. Combined with immunohistochemistry, these were consistent with large cell neuroendocrine carcinoma. The tumor in the common bile duct was a poorly differentiated carcinoma, with a maximum diameter of approximately 1 cm, surrounded by lymphoid tissue and lymphatic sinuses. This was considered to represent metastatic large cell neuroendocrine carcinoma within a lymph node, with extranodal extension. Tumors were identified at multiple sites in the patient. Based on imaging findings and postoperative assessment, the tumor growth pattern was most suggestive of a primary mixed adenocarcinoma-large cell neuroendocrine carcinoma originating in the gallbladder fundus, with metastatic large cell neuroendocrine carcinoma to the gallbladder neck, liver, porta hepatis, and common bile duct region. All surgical margins, including the pancreatic stump margin, gastric stump margin, duodenal stump margin, liver margin, bilateral margins, portal vein margin, and gastrocolic omentum, were negative for carcinoma. The liver contained both a direct invasion mass (segment V) and separate metastatic nodules in segments IVb etc., confirming M1 stage. Diagnosis: Gallbladder malignancy (pT4N1M1, Stage IVB). Postoperatively, the patient was transferred to the ICU for anti-infection and nutritional support treatment. After 5 days, she was transferred to a regular ward. She continued to receive enteral nutrition support and acupuncture to promote gastrointestinal function recovery. After one month, the patient’s condition was essentially stable and improved, and she was discharged at her request. The patient was followed for 8 months post-surgery. Serial imaging showed no local recurrence or distant metastases. She refused adjuvant chemotherapy. She elected for clinical and radiological surveillance.

## Discussion

This case presents a rare gallbladder malignancy that was challenging to diagnose and complex to treat. The patient initially presented due to an incidental finding, which underscores the typically asymptomatic nature of gallbladder cancer until it progresses to an advanced stage (1). The significantly elevated CEA level, while not specific for neuroendocrine neoplasms, served as a key indicator of malignancy. Imaging findings were highly suggestive of advanced disease with direct liver invasion and lymph node metastases, which was subsequently confirmed by histopathology.

The definitive diagnosis of mixed approximate 60% adenoma- 40% large cell neuroendocrine carcinoma was established through postoperative immunohistochemistry. Positive results for synaptophysin (Syn) confirmed the neuroendocrine phenotype (3), while the high Ki-67 index (approximately 80%) classified it as a high-grade neuroendocrine carcinoma according to the WHO grading system. The coexistence of an adenocarcinoma component alongside large cell neuroendocrine carcinoma classifies this tumor as a mixed neuroendocrine-non-neuroendocrine neoplasm (MiNEN), a phenomenon recognized in other organs but exceedingly rare in the gallbladder (2, 4). This mixed lineage suggests the possible existence of a common precursor cell capable of divergent differentiation (5, 6), which has significant implications for tumor behavior and potential therapeutic targets, as each component may respond differently to systemic therapy (6).

For stage IVB gallbladder mixed adenoma-large cell neuroendocrine carcinoma, the prognosis is typically poor due to its high-grade nature and advanced stage at diagnosis (3). Surgical resection remains the cornerstone of potentially curative treatment for gallbladder cancer (1). The en bloc resection performed in this case, including partial hepatectomy, pancreaticoduodenectomy (to ensure adequate clearance of the extrahepatic bile duct and lymphatic tissue), and partial portal vein resection with reconstruction, addressed the extensive local invasion and lymph node involvement. All detected lesions were encompassed in the extended resection. No unresectable distant metastases were present. Successful vascular reconstruction ensured hepatic blood flow, a critical factor for patient safety and recovery. Despite extensive disease, achieving negative surgical margins may be a feasible treatment option in carefully selected patients; however, the oncological benefit remains to be validated. This case highlights the importance of a multidisciplinary approach involving surgeons, pathologists, and oncologists in managing such complex and rare tumors, and the need to balance the benefits of radical surgery against its significant associated risks (3).

The presented case demonstrates the optimal outcome achievable when patients have access to a multidisciplinary team, advanced surgical expertise, and comprehensive postoperative care. However, it also implicitly highlights the potential inequalities in surgical oncology. For a patient with the same stage IVB gallbladder MiNEN but residing in a resource-limited setting—without access to routine health screening, a specialized hepatobiliary center, or intensive postoperative rehabilitation—the prognosis would likely be drastically different. This disparity in access (to early detection and specialized centers), experience (lack of MDT discussion), recovery (inadequate nutritional support and rehabilitation), and ultimately outcomes underscores the critical need for strategies to bridge these gaps in cancer care globally.

## Conclusion

This case report describes a rare case of mixed adenocarcinoma-large cell neuroendocrine carcinoma of the gallbladder, which presented with locally extensive progression (pT4N1M1, stage IVB) upon incidental discovery during a routine physical examination. This case highlights the high complexity of diagnosing such tumors, with definitive diagnosis relying entirely on postoperative pathological and immunohistochemical analysis (Syn+, CK19+, Ki-67+ approximately 80%, INSM1+, and CgA+ approximately 10%), and reveals its characteristic biphenotypic differentiation comprising both adenocarcinoma and high-grade neuroendocrine carcinoma components. Faced with this aggressive advanced tumor lacking standardized treatment protocols, this case underwent precise evaluation and decision-making by a multidisciplinary team (MDT), leading to the implementation of extended radical en bloc resection including partial hepatectomy, pancreaticoduodenectomy, and portal vein resection with reconstruction, successfully achieving negative margins (R0 resection) in all specimens. The clinical value of this case lies in: firstly, providing new insights into the clinicopathological characteristics of this ultra-rare gallbladder mixed large cell neuroendocrine carcinoma; secondly, demonstrating that under careful patient selection and multidisciplinary collaboration, even for locally advanced MiNEN, implementing extended radical surgery to achieve R0 resection is a potential treatment option. While R0 resection was achieved, the oncological benefit of this aggressive approach in stage IVB disease requires longer follow-up and further validation.

## Data Availability

The original contributions presented in the study are included in the article/supplementary material. Further inquiries can be directed to the corresponding authors.
